# COVID-19 Control: Can Germany Learn From China?

**DOI:** 10.34172/ijhpm.2020.78

**Published:** 2020-05-27

**Authors:** Olaf Müller, Guangyu Lu, Albrecht Jahn, Oliver Razum

**Affiliations:** ^1^Institute of Global Health, Medical School, Ruprecht-Karls-University, Heidelberg, Germany; ^2^Department of Public Health, Medical College, Yangzhou University, Yangzhou, China.; ^3^Department of Epidemiology & International Public Health, School of Public Health, Bielefeld University, Bielefeld, Germany.

**Keywords:** SARS-CoV-2, Pandemic, Control, Lock Down, Health Policy

## Abstract

The coronavirus disease 2019 (COVID-19) outbreak started in China in December 2019 and has developed into a pandemic. Using mandatory large-scale public health interventions including a lockdown with locally varying intensity and duration, China has been successful in controlling the epidemic at an early stage. The epicentre of the pandemic has since shifted to Europe and The Americas. In certain cities and regions, health systems became overwhelmed by high numbers of cases and deaths, whereas other regions continue to experience low incidence rates. Still, lockdowns were usually implemented country-wide, albeit with differing intensities between countries. Compared to its neighbours, Germany has managed to keep the epidemic relatively well under control, in spite of a lockdown that was only partial. In analogy to many countries at a similar stage, Germany is now under increasing pressure to further relax lockdown measures to limit economic and psychosocial costs. However, if this is done too rapidly, Germany risks facing tens of thousands more severe cases of COVID-19 and deaths in the coming months. Hence, it could again follow China’s example and relax measures according to local incidence, based on intensive testing.

## Introduction

In December 2019, a pandemic of coronavirus disease 2019 (COVID-19) began, caused bythe severe acute respiratory syndrome virus (SARS-CoV-2).^1-3^ The World Health Organization (WHO) declared the COVID-19 outbreak as a *Public Health Emergency of International Concern* on January 31, 2020, but classified it as a pandemic only by March 11.^4^ As of May 19, a total of 4.8 million confirmed cases, including 320 000 deaths, have been reported from 188 countries.^5^ By then, Europe and The Americas had become the new epicentres of the COVID-19 pandemic, with several times more SARS-CoV-2 infections and COVID-19 deaths compared to China.^5^

## The Outbreak in China

**Figure 1:**
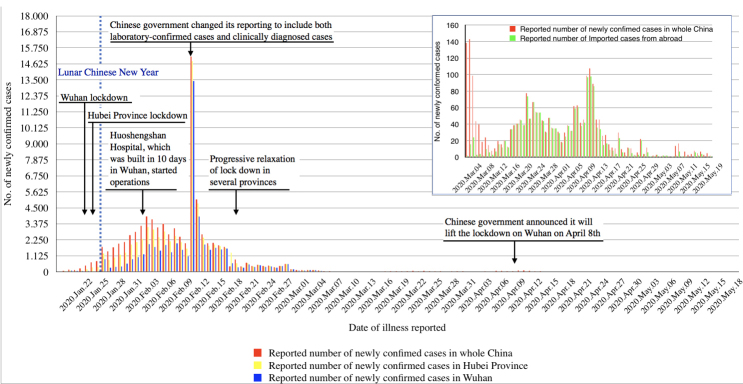
COVID-19 Control Interventions in China and Development of Laboratory-Confirmed Cases From January 20 to May 19, 2020. Abbreviation: COVID-19, coronavirus disease 2019.

China, where the outbreak was first detected, experienced an exponential growth of confirmed COVID-19 cases in January 2020.^6^ The government reacted rapidly and implemented massive public health interventions, in particular a combination of a range of social distancing and other established non-pharmaceutical epidemic control measures.^7,8^ By January 23, an intense lockdown was implemented in Wuhan, the early epicentre of the epidemic, followed by Hubei province and finally nearly all of China.^7^ The lockdown, together with specific interventions in Wuhan such as construction of new hospitals and employment of several thousand medical doctors from other parts of China, constituted the *“perhaps most ambitious, agile and aggressive disease containment effort in history.”*^8^ Subsequently, the number of reverse transcription polymerase chain reaction (RT-PCR)-confirmed newly reported COVID-19 cases declined substantially within 4 weeks in the whole of China, and the number of new autochthonous infections approached zero in early March 2020 (Figure 1).^9,10^ Given these developments, control intensities have been progressively relaxed in Chinese provinces since February 17, at a time when the instantaneous effective reproduction number (Rt) had clearly declined below one.^11^ While from mid-March until the end of April all newly confirmed COVID-19 cases were reportedly imported, new autochthonous cases occurred in Wuhan and in a few other Chinese cities in May, which are now subject to intense local control measures (Figure 1).

## The Situation in Germany

**Figure 2:**
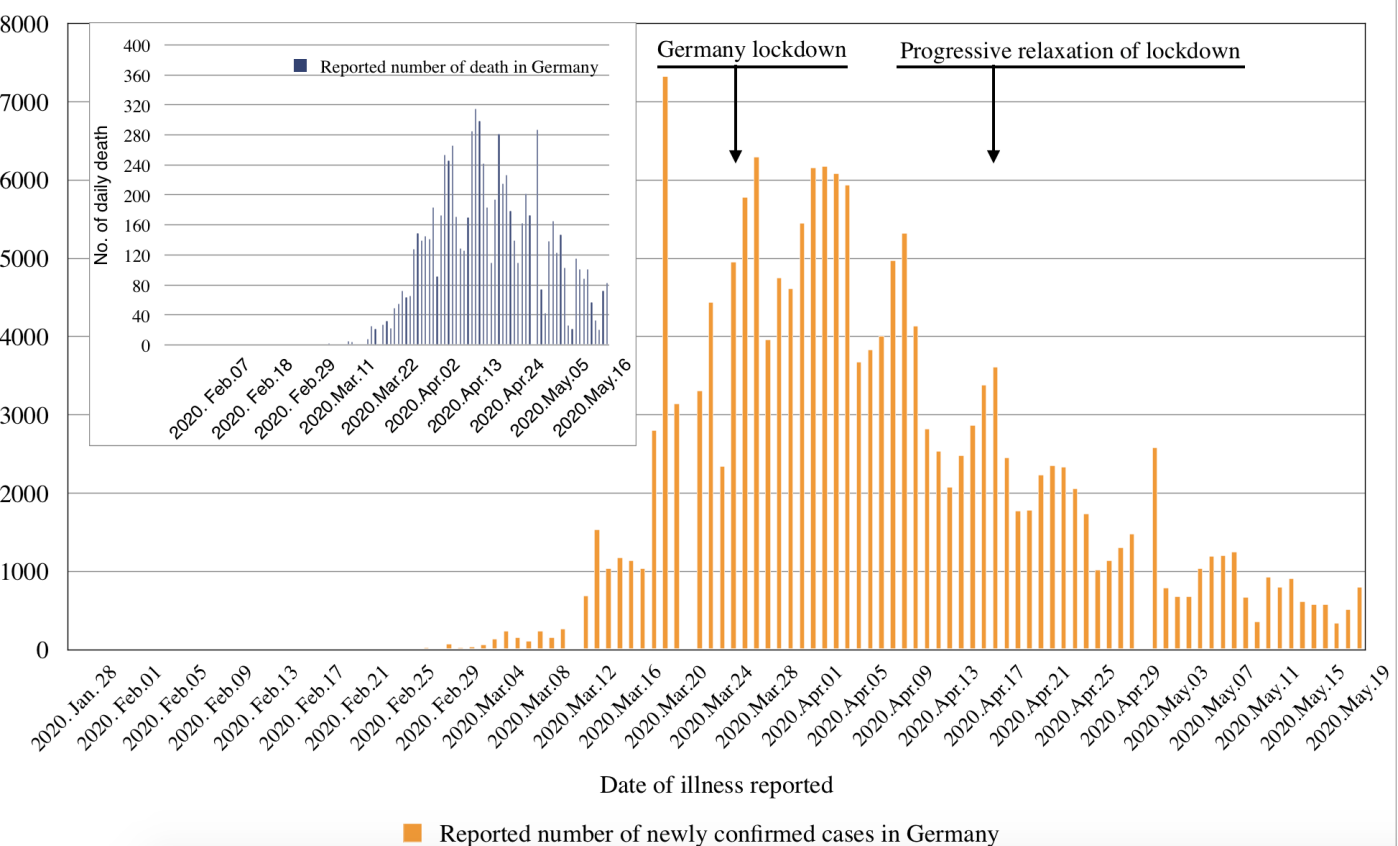
COVID-19 Control Interventions in Germany and Development of Laboratory-Confirmed Cases From January 28 to May 19, 2020. Abbreviation: COVID-19, coronavirus disease 2019.

In Germany, first cases of COVID-19 were seen by the end of January 2020.^12,13^ By May 19, Germany reported a total of 175 210 RT-PCR-confirmed SARS-CoV-2 infections, including 8007 deaths, which is among the highest number of cases in Europe.^14^ However, a rather low case fatality rate is observed in Germany in comparison to other European countries with a high number of reported COVID-19 cases (Italy, Spain, France, the United Kingdom), which could be explained by an early and broad testing strategy, an initially younger SARS-CoV-2 infected population due to transmission at carnival meetings and in ski resorts, and a high-capacity, high-standard healthcare system.^4,14-18^ Moreover, COVID-19 incidence varies largely between the states in Germany, with the highest disease burden being reported from the southern states Bavaria and Baden-Württemberg.^14^ In view of the rapidly rising case-load and the high case fatality rate in neighbouring countries, a partial lockdown was implemented and largely respected in Germany on March 22, initially until April 19. Compared to the measures taken in China, as well as later in Italy and Spain, the German lockdown can be considered as moderate with people still having been able to move about individually rather freely.^4^ Still, the results look promising; this may have to do with a rather good compliance likely supported by the daily broad and fact-oriented discussion of national and international pandemic developments in the German media. The number of newly detected COVID-19 cases has consistently declined since early April (Figure 2).^14^ On April 15 – when there were still a few thousand new SARS-CoV-2 infections reported every day – the German Government announced an extension of the partial lockdown until May 3, but with a number of relaxing elements to reduce psychosocial consequences and harm to the economy. Experts now worry that, with a Rt of about one at the time of the decision and not much reduced since, a too liberal relaxation may lead to a rapid resurgence of COVID-19 cases.^18,19^ A further relaxation has already started on May 3, and the intense and controversial discussion about the precise course of a safe exit strategy for Germany continues.

## Global Developments

In the absence of effective biomedical interventions, the global response to the COVID-19 pandemic has been the employment of a combination of containment and mitigation activities.^4,20^ The main goals of these public health interventions were to flatten the epidemiological curve, to protect the high risk groups of elderly people and patients with chronic diseases, and in particular to prevent an overwhelming of clinical services.^21^ National responses varied widely, ranging from early and intensive surveillance measures, coupled with aggressive approaches to find cases and their contacts and to isolate and quarantine them (eg, in Taiwan, South Korea, Singapore), to a more *laissez faire* approach sometimes even with the idea to rapidly achieve herd immunity as it was initially the case in the United Kingdom and as continues to be the case in Sweden.^22-25^ However, with the observation of a rapid overwhelming of existing hospital and intensive care unit capacities and corresponding high deaths rates in Europe, which started in northern Italy and later occurred in areas of Spain, France and the United Kingdom, as well as the United States, the majority of countries worldwide implemented lockdowns.^16,25,26^ Consequently, there is now an intense discussion on the best exit strategies.^26^

## Policy Alternatives for Germany

Germany needs to choose a strategy now that will probably lie somewhere in-between two options at either end of a range: At one extreme, it could follow the example of China and continue the lockdown until the number of SARS-CoV-2 infections is brought close to zero; the strategy should then be to maintain infection rates at very low levels until a vaccine becomes available.^27^ This would likely be possible through combining further increased testing for SARS-CoV-2 in the population with intensified contact-tracing followed by systematic isolation and quarantine measures. Such a strategy would later enable a rational relaxation of control measures and re-opening of institutions (eg, schools) as has already been successful in countries like China, Singapore, Taiwan, South Korea, and New Zealand.^22,27-29^ The economic and psychosocial cost of a prolonged lockdown could be very high, however.^26^ At the other extreme, with slightly relaxing measures now, Germany might manage to keep the epidemic at its present level, which currently looks promising in particular when compared to the statistics of its neighbours (Figure 2).^14^ There is a price as well: the German daily COVID-19 death figures were already high in March and April and – although they have substantially decreased since – there remains a certain risk of further substantial mortality given the ongoing spread of the virus into older population groups, in particular into the populations of retirement homes and inhabitants of long-term-care facilities.^30,31^ A resurgence of COVID-19 in Germany could result in a cumulative total of tens of thousands of deaths over the following months. This may be considered as unacceptable and also unethical, even if the German health system would be able to cope with such high case numbers.^4,14,18,26^

Current strategies for COVID-19 control need to be continuously adjusted, and new combinations of non-medical preventive interventions appear to be promising. Besides physical distancing and hygiene procedures, use of face masks for healthy individuals in the community is increasingly considered as a potentially feasible and effective intervention to control respiratory viruses including SARS-CoV-2.^32^ A policy for mandatory face masking in public buildings and public transport has also been intensively discussed in Germany and is currently being adopted in the whole country. FFP2/3 masks and medical masks should continue to be reserved for professional health staff, but cloth masks could easily be produced for mass masking during the pandemic.^32,33^ While there has been some doubt and reservation regarding the effectiveness of mass masking, there is now increasing support for a likely benefit of such an intervention both in Germany and worldwide.^34-37^ Even more importantly, given the obvious local differences in the intensity of COVID-19 incidence, control measures should be adjusted to local circumstances, in line with the German political constitution.^38^ However, there needs to be a fine balance between actions of individual states and communities in Germany on the one hand and the federal Government on the other to avoid contradictory messages to the population.

Other strategies that have been proposed lack an evidence-base and seem socially unacceptable. Predominantly, this applies to isolating all elderly persons and people with chronic diseases while allowing younger and healthy members of society to get infected and to develop immunity (leading also to the development of herd immunity). It seems unrealistic that it would be possible to protect the large at-risk population over a prolonged period of time from getting infected. Moreover, COVID-19 is causing severe disease and death even in a small proportion of previously healthy middle-aged adults.^8,26,39^

## Conclusions

China’s epidemic management provides an important experience from which countries such as Germany can learn. This is of particular importance as an early availability of game-changing drugs against COVID-19 appears to be more and more illusionary and as the availability of an effective vaccine will likely need another one to two years.^40,41^ As all countries which are currently under lockdown are facing the same challenge to keep SARS-CoV-2 infection numbers low after relaxing the control measures, the world may be able to learn again from the ongoing experience with such an exit strategy in China.^26,27^ This applies in particular to Germany, which would risk to lose many of its achievements in case of a severe second wave of the epidemic. There is a clear limit though: In contrast to China, Germany should rely on its established tradition of political and societal dialogue. It should continue to involve not only virologists and epidemiologists, but social scientists and all population segments when making the painful choices that are required to keep the epidemic under control.

## Ethical issues

Not applicable.

## Competing interests

Authors declare that they have no competing interests.

## Authors’ contributions

All authors contributed to the text, read, and approved the final manuscript.

## Authors’ affiliations

^1^Institute of Global Health, Medical School, Ruprecht-Karls-University, Heidelberg, Germany. ^2^Department of Public Health, Medical College, Yangzhou University, Yangzhou, China. ^3^Department of Epidemiology & International Public Health, School of Public Health, Bielefeld University, Bielefeld, Germany.
